# Multi-Functional Repair and Long-Term Preservation of Paper Relics by Nano-MgO with Aminosilaned Bacterial Cellulose

**DOI:** 10.3390/molecules29163959

**Published:** 2024-08-22

**Authors:** Hongyan Mou, Ting Wu, Xingxiang Ji, Hongjie Zhang, Xiao Wu, Huiming Fan

**Affiliations:** 1 State Key Laboratory of Pulp and Paper Engineering, School of Light Industry and Engineering, South China University of Technology, Guangzhou 510640, China; 2 Key Laboratory of Pulp and Paper Science & Technology of Ministry of Education, Qilu University of Technology (Shandong Academy of Sciences), Jinan 250353, China; xxjt78@163.com; 3 National Engineering Laboratory for Pulp and Paper, Beijing 100102, China

**Keywords:** paper relics, conservation, bacterial cellulose, nano-MgO, aging resistance

## Abstract

Paper relics, as carrieres of historical civilization’s records and inheritance, could be severely acidic and brittle over time. In this study, the multi-functional dispersion of nanometer magnesium oxide (MgO) carried by 3-aminopropyl triethoxysilane-modified bacterial cellulose (KH550-BC) was applied in the impregnation process to repair aged paper, aiming at solving the key problems of anti-acid and strength recovery in the protection of ancient books. The KH550-BC/MgO treatment demonstrated enhanced functional efficacy in repairing aged paper, attributed to the homogeneous and stable distribution of MgO within the nanofibers of BC networks, with minimal impact on the paper’s wettability and color. Furthermore, the treatment facilitated the formation of adequate alkali reserves and hydrogen bonding, resulting in superior anti-aging properties in the treated paper during prolonged preservation. Even after 30 days of hygrothermal aging tests, the paper repaired by KH550-BC/MgO was still in a gently alkaline environment (pH was about 7.56), alongside a 32.18% elevation compared to the untreated paper regarding the tear index. The results of this work indicate that KH550-BC/MgO is an effective reinforcement material for improving the long-term restoration of ancient books.

## 1. Introduction

Paper literature has an incalculable significance and serves as a vital conduit for historical civilization in many eras and nations [1]. Affected by the pulp and paper technology and the harsh storage environment, a large number of documents and books are irreversibly acidified and aged, seriously affecting the service and storage life [2,3,4,5]. In recent years, countries around the world have begun to pay attention to and explore the research topic of the protection and restoration of ancient books. The previous repair process typically involved a series of step-by-step processes, including deacidification, reinforcement, and antibacterial and mold prevention [6,7]. These processes were complex and inefficient and could easily cause secondary damage to the paper. Therefore, multi-functional methods for restoring ancient books have become a key research direction [8,9,10,11].

Bacterial cellulose (BC), as a green natural polymer material, has an ultra-fine three-dimensional porous fiber structure and excellent mechanical properties [12,13], offering great prospects in the reinforcement and protection of paper documents [14,15,16]. In our previous study, BC-repaired paper exhibited a high folding endurance of 28 times, indicating a promising application of BC as a restoration material [17]. However, the long-term stability of BC for the preservation of ancient books is limited by its hydrophilicity and the fact that BC cannot be deacidified [18]. The unique chemical composition of BC, which is rich in hydroxyl groups, offers a promising avenue for the development of new materials with more functionalities [19]. For instance, mineralized BC had been successfully employed in the deacidification of paper-based materials, while simultaneously imparting paper with exceptional flame retardant properties [20]. Another reliable approach for the repurposing of BC is amino silanization [21,22,23]; the results of our latest research indicated that this process improves the interface bonding between BC and aged paper toward higher mechanical properties of the treated paper [24].

Furthermore, adequate alkali reserves are usually considered as a guarantee of long-term acid resistance in paper [25]. Recently, alkaline nanoparticles have been the favored deacidifying materials because of their smaller particle size and better permeability, which helps to further extend the durability of paper [26,27]. From a more positive perspective, nano-magnesia is regarded by conservation scientists as a preferred deacidifier among magnesium derivatives, primarily due to its milder alkalinity compared to magnesium hydroxide. However, an inherent limitation of nano-MgO is its tendency to settle out in the solvent because of its poor dispersion stability. Oleic-acid-modified nano-MgO was used to promote its dispersion stability in a cyclohexane non-polar solvent for paper deacidification [28]. He et al. [29] also created a stable organic covering that allowed for the uniform deposition of nano-MgO particles on paper, based on the co-dispersion of trimethylsilyl cellulose (TMSC) and isopropyl alcohol (IPA). Our theory posits that the long-chain network structure of KH550-BC could impede the agglomeration of nano-MgO particles through steric hindrance while enhancing the alkalinity of KH550-BC, which is beneficial for the long-term stability of KH550-BC/MgO-reinforced paper.

Herein, a nano-composite dispersion of KH550-BC and MgO was prepared and developed by adhering to the principle of “repairing old as before”. The repair effect and anti-aging properties of aged paper were improved using KH550-BC/MgO, and the process was comprehensively investigated. The anti-aging mechanism of KH550-BC/MgO to aged paper was studied via ATR-Fourier infrared spectroscopy (ATR-FTIR) and X-ray diffraction spectroscopy (XRD), and the distribution and deposition of KH550-BC/MgO in paper were analyzed through field-emission scanning electron microscopy (FESEM) as a supplement for evaluating the long-term stability of KH550-BC/MgO-reinforced aged paper.

## 2. Results and Discussion

### 2.1. Effect of Loading Nano-MgO in KH550-BC on Deacidification

In the KH550-BC/MgO system, the addition of nano-MgO should give the paper enough alkali storage to extend the service life of the paper, but pH and alkali storage that are too high would cause the alkaline degradation of the paper fiber and yellowing of the paper after deacidification [30,31,32]. To reduce these undesirable changes, we investigated the effect of nano-MgO at varying concentrations, and the results were compared with KH550-BC.

**Figure 1:**
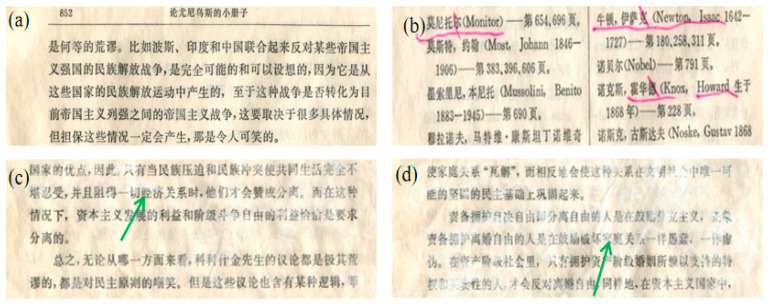
The influence of MgO contents in the KH550-BC/MgO system on the appearance of the paper samples, wherein (**a**) represented the paper repaired by KH550-BC, (**b**–**d**) represented the paper repaired by KH550-BC/MgO, in which the MgO content was (**b**) 0.1%, (**c**) 0.2%, or (**d**) 0.3%.

As shown in Table 1, with an increase in MgO content in the KH550-BC/MgO system, the pH value of the repaired paper significantly increased, and the alkali reserves, as the key factor for the anti-acidification of paper, exceeded 0.30 mol/kg. In images of paper repaired by KH550 loading different contents of MgO, the MgO at 0.1% could distribute on paper evenly, as shown in Figure 1b. However, at concentrations above 0.1% (as the green arrows pointed), MgO with a high specific surface energy was prone to agglomeration and deposition on the paper surface. As a consequence, 0.1% MgO was chosen to be appropriate for paper restoration in accordance with the lack of interference with paper font ink. At this point, the grammage of the paper was increased by 3.25 g/m^2^, and its alkali reserve was 0.36 mol/kg, which was 41.18% higher than that of KH550-BC repair alone (0.26 mol/kg). 

### 2.2. Strengthening Effect of KH550-BC/MgO on Aged Paper 

While KH550-BC/MgO had the better deacidification effect on aged paper (Table 1), the strengthening effects of KH550-BC/MgO on aged paper were also maintained at a high level (Table 2). P2 outperformed the reference sample (UP) in terms of the tear index, tensile index, and folding endurance, with values of 6.60 mN m^2^/g, 35.44 N m/g, and 19 times, respectively. In addition, the impregnation treatment with KH550-BC/MgO resulted in a 13.91% increase in the zero-span tensile strength, while the tensile stress also increased by 88.60%. It could be indicated that KH550-BC/MgO has significantly improved the strength of the individual fibers and the tensile performance. All of these phenomena may be induced by the hydrogen bonds between modified bacterial cellulose being opened, exposing the free hydroxyl groups on its molecules, which form more hydrogen bonds with paper fibers, thereby increasing the binding force between fibers of aged paper. Meanwhile, some BC was adsorbed on the surface of fibers, interweaving and winding to form a network structure, thereby increasing the strength of each paper fiber [17]. This hypothesis will be verified by subsequent analysis.

### 2.3. Wettability Analysis of Repaired Paper

**Figure 2:**
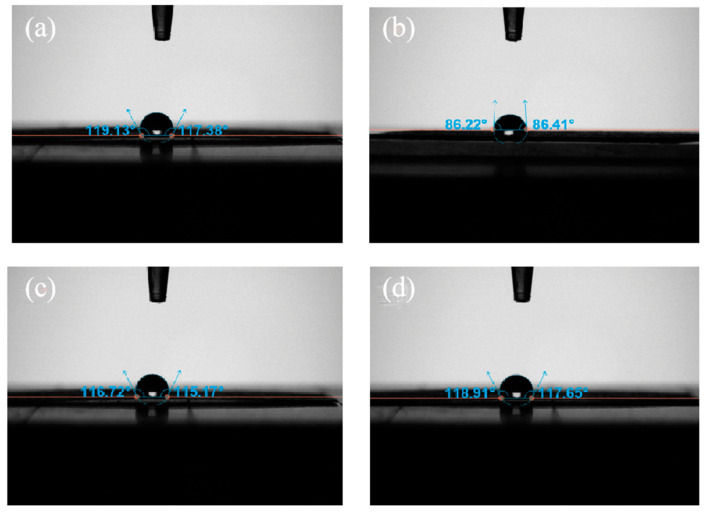
The water contact angles of (**a**) the untreated paper and (**b**–**d**) the paper repaired by BC, KH550-BC, and KH550-BC/MgO.

Following the repair with KH550-BC/MgO, Figure 2 shows that the contact angle between the paper and water was 118°, which was nearly identical to that of the base paper UP (119°). The hydrophobicity of UP was correlated with the chemical agent used in the papermaking process. In order to prevent the paper from being damaged by wetting, sizing agents were added to enhance the paper’s resistance to liquid penetration and diffusion ability [33,34,35]. However, as shown in Figure 2b, the addition of BC, which contained a large number of hydrophilic hydroxyl groups, rendered the paper susceptible to deterioration when exposed to humid conditions over an extended duration following the repair process, due to the intrinsic hygroscopic nature of BC [36]. This weakness was improved by modifying BC with the inclusion of nano-MgO [13]. Specifically,, the surface effect of nano-MgO counterbalanced the hydrophilic effect of BC, resulting in a rougher surface and decreased porosity of the paper. As a result, the repaired paper was able to retain its hydrophobicity, ensuring its preservation in various environments for extended periods of time.

**Figure 3:**
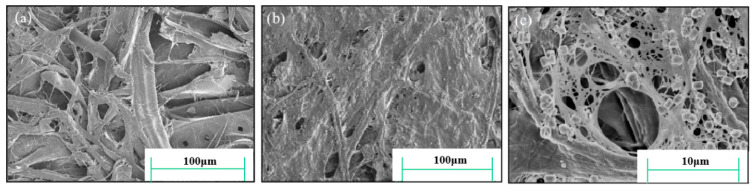
FESEM images of (**a**) the untreated paper and (**b**,**c**) the paper repaired by KH550-BC/MgO, which were magnified by (**b**) 500× and (**c**) 5000×.

Furthermore, as illustrated in Figure 3, the SEM images of the paper prior to and following the KH550-BC/MgO repair process confirmed that KH550-BC/MgO effectively covered the fiber surface and bridged between the fiber, resulting in a notable enhancement of the paper’s mechanical properties and a reduction in its porosity (Figure 3a,b). At 5000× magnification, the stable dispersion and uniform adsorption of nano-sized MgO in the three-dimensional fiber network of BC were clearly observed in Figure 3c, demonstrating the stable deacidification of KH550-BC/MgO and its potential for durability.

### 2.4. Aging Resistance and Stability Studies of Repaired Paper

To further confirm the fact that KH550-BC/MgO improves the anti-aging properties of paper, artificial aging tests were carried out on aged paper before and after repair. The changes in the physical strength, acid–base property, and color appearance of all of the repaired paper samples during artificial aging were meticulously analyzed. 

#### 2.4.1. Mechanical Properties Change in Repaired Paper during Aging 

**Figure 4:**
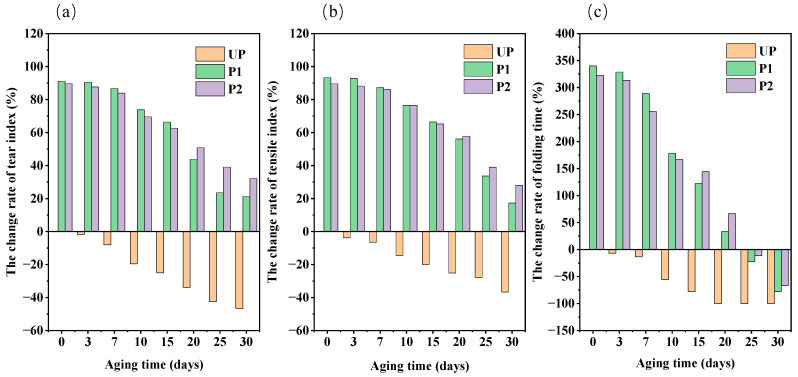
The change rate of the paper samples strength properties with aging time, in which (**a**) represented the tear index, (**b**) represented the tensile index, and (**c**) represented the folding endurance.

A thorough investigation was conducted into the variations in the strength of the paper reinforced with KH550-BC and KH550-BC/MgO as they aged for different days. The results are presented in Figure 4. Given that the untreated paper had an acceptable initial strength (tear index of 3.48 mN·m^2^/g, tensile index of 18.70 N·m/g, folding endurance of 4.5 times), there was no discernible decrease in the strength of any of the repaired paper samples after 3 days of artificial aging.

As illustrated in Figure 4a, during the aging period from 3 d to 15 d, the tear index of the untreated paper (UP) dropped sharply by 25.00%. This decline was attributed to the accumulation of acid rather than its consumption over time, leading to the constant acid degradation of cellulose in UP. Relevant research has also demonstrated a positive correlation between the concentration of H^+^ and the rate of cellulose hydrolysis [37,38]. In comparison to the initial tear index of UP, the tear index of P1 and P2 after 15 days of aging was 66.38% and 62.64% higher, respectively. The enhancement effect of KH550-BC/MgO was not only reflected in the significant improvement of the interfacial adhesion between the BC and the aged paper, but more importantly, it provided enough of an alkali reserve for the repaired paper to continue to consume acidic substances produced in high-temperature and high-humidity environments. Therefore even after 30 d of aging, the tear index of P2 (4.60 mN·m^2^/g) was higher than that of P1(4.22 mN·m^2^/g), and it was still 32.18% higher than that of UP. The MgO present in KH550-BC acts as a strengthening agent, significantly enhancing the paper’s anti-aging ability and prolonging its conservation duration.

The tensile index changes in all paper samples are shown in Figure 4b. On the 15th day of aging, the tensile index of UP decreased by 19.84%. Simultaneously, the paper enhanced by KH550-BC and KH550-BC/MgO dispersion showed a slight decrease, yet still retained a higher tensile index than UP, at 66.42% and 65.24%, respectively. The tensile index of P2 progressively surpassed that of P1 during the aging process of 15–20 days, and its reduction was the least during the whole aging period, which also indicates that the paper samples treated with KH550-BC/MgO had a good aging resistance stability [30].

Nonetheless, the changes in folding endurance in the aging experiment were noteworthy (Figure 4c). After 15 days of aging, the folding endurance of UP decreased by 77.78%, from 4.5 times to 1 time, while P1 demonstrated a 49.49% decrease, and P2 exhibited a 42.11% reduction. Compared to the tear index and the tensile index, the folding endurance images of KH550-BC- and KH550-BC/MgO-treated paper intersected between 10 and 15 days, which meant that the folding endurance of the paper was more susceptible to environmental pH and alkali storage [39]. All the above results underscored that the anti-aging property of paper can be effectively enhanced by KH550-BC/MgO.

#### 2.4.2. Resistance to Acidification of Repaired Paper 

**Figure 5:**
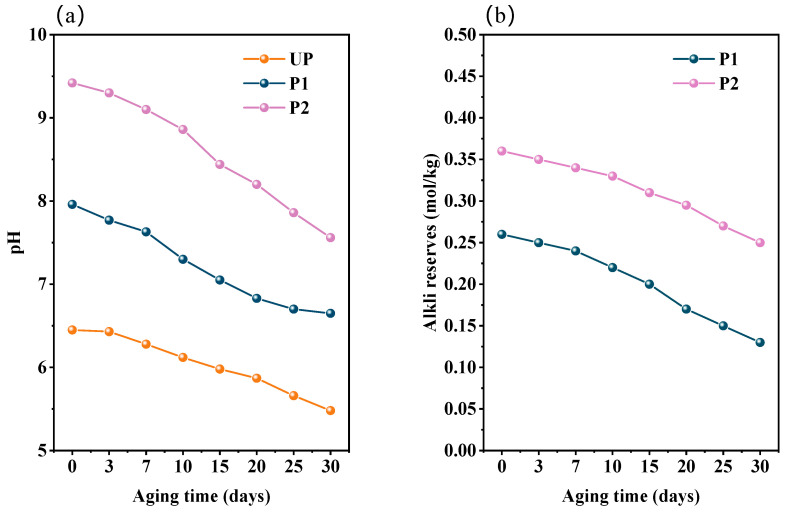
The change curves of (**a**) the pH and (**b**) the alkali reserves of paper samples with aging time.

The pH value and alkali reserve were the important indicators for evaluating the deacidification effect of aged paper samples repaired by KH550-BC and KH550-BC/MgO. The environment of high temperature and humidity accelerated the degradation of the primary components of the paper, resulting in the accumulation of acidic substances [40,41,42]. This was the main factor contributing to the observed decline in the pH value across all paper samples (Figure 5). In the 30-day artificial aging process, the pH of P1 was gradually changed from 7.96 to 6.65, confirming that the amino groups of KH550-BC exerted a buffer effect on the degradation of paper fibers. However, P2 remained weakly alkaline after 30 days of aging, with a pH value of 7.56 and an alkali reserve of 0.25 mol/kg. This further indicated that the fiber network structure of BC stabilized the nano-MgO and played a positive role in covering [43], thus allowing for a gradual and continuous process of resisting acidification compared to KH550-BC. 

#### 2.4.3. Whiteness and Chromatic Aberration Change during Aging 

**Figure 6:**
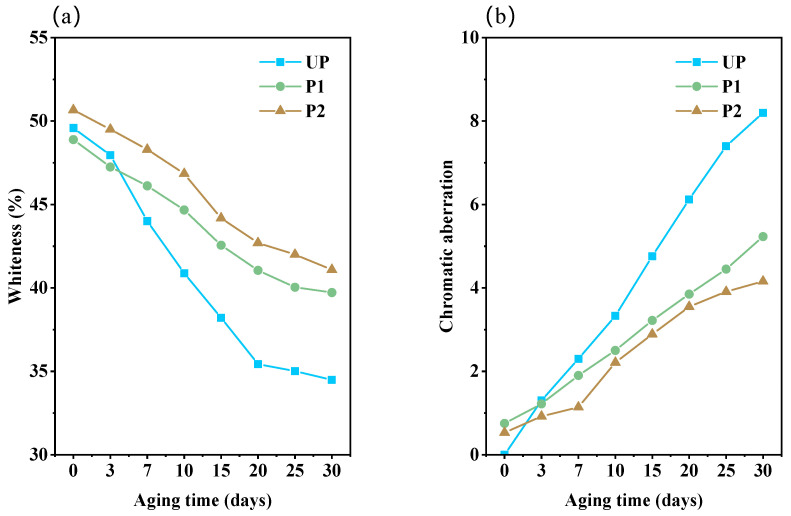
The change curves of (**a**) whiteness and (**b**) chromatic aberration of paper samples with aging time.

**Figure 7:**
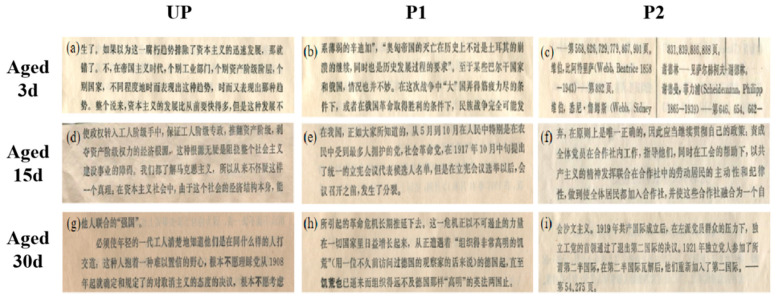
The scanning images of the paper samples with aging time, wherein (**a**,**d**,**g**) represented UP, (**b**,**e**,**h**) represented P1, and (**c**,**f**,**i**) represented P2.

Consistent with the above changes in mechanical properties and pH properties, aging for 3 days had a negligible impact on the whiteness and color difference of all paper samples (Figure 6). More chromophoric groups were formed during the accelerated lignin degradation in the acidifying UP throughout the hygrothermal aging process which was the cause of the significant yellowing observed in UP [37]. The whiteness decreased from the initial 49.59% to only 34.49%, along with the color difference increased to 8.20 after 30 days of aging. In contrast, the whiteness of P2 treated with KH550-BC/MgO exhibited the slowest decrease, as evidenced by a postponed aging process and a decreased yellowing rate due to the presence of nano-MgO [44]. Aged for 30 days, the values of whiteness and color difference were 41.09% and 4.16, respectively (Figure 6). What is more, the macroscopic scanning images of all of the paper samples (Figure 7) during the aging process corresponded to the change trends of whiteness and chromatic aberration as mentioned earlier, also illustrating that KH550-BC/MgO dispersion was the optimal repair system.

### 2.5. Anti-Aging Mechanism Analysis

**Figure 8:**
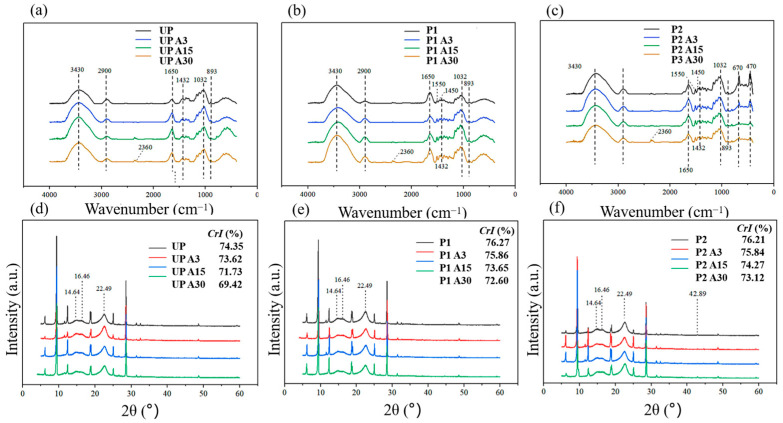
FTIR-transform infrared spectra (**a**–**c**) and X-ray diffraction patterns (**d**–**f**) of the paper samples, wherein (**a**,**d**) represented UP, (**b**,**e**) represented P1, and (**c**,**f**) represented P2.

ATR-FTIR spectra were performed on each of the three paper samples to analyze the changes in the functional groups during the aging process [45]. As shown in Figure 8a–c, after repair by KH550-BC and KH550-BC/MgO, the C=O stretching vibration peak of UP at 1650 cm^−1^ was transferred to 1641 cm^−1^ and 1644 cm^−1^, thereby confirming the existence of hydrogen bonding between the reinforcing agent and the aging paper fiber [46]. New peaks emerged at 1550 cm^−1^ and 1450 cm^−1^, which were primarily attributed to the bending vibration of NH_2_ and the stretching vibration of C-N in KH550-BC. This indicated that the modified BC was successfully combined with the paper fibers [24]. Furthermore, in Figure 8c, peaks corresponding to Mg-O stretching vibration and bending vibration were evident at 670 cm^−1^ and 470 cm^−1^, respectively. The strength of the characteristic peak of MgO decreased throughout the aging process, suggesting that MgO continued to exert an anti-acidifying effect during this period.

The degree of aging of the different papers can be compard by exploring the crystallinity index of the cellulose in Figure 8d–f [47,48,49]. Following the application of the two repair systems, the CrI of P2 (76.21%) was marginally higher than that of UP (74.33%) comparable to that of P1 (76.25%). These findings were consistent with the results of the mechanical property analysis (Table 2). With the gradual aging of the paper, the decrease rate of the crystallinity of P1 was lower than that of UP (Figure 8d,e), suggesting that P1 had a certain aging resistance provided by the high strength and favorable interface bonding of KH550-BC. Based on these observations, Figure 8f illustrated that the CrI of P2 exhibited a minimal variation; after aging for 30 d, the CrI was 73.12%, with a reduction of 3.09%. The slowest rate of reduction in the cellulose crystallization index demonstrated the inhibition of P2 aging and the durability of KH550-BC/MgO’s protective action [50,51]. 

**Figure 9:**
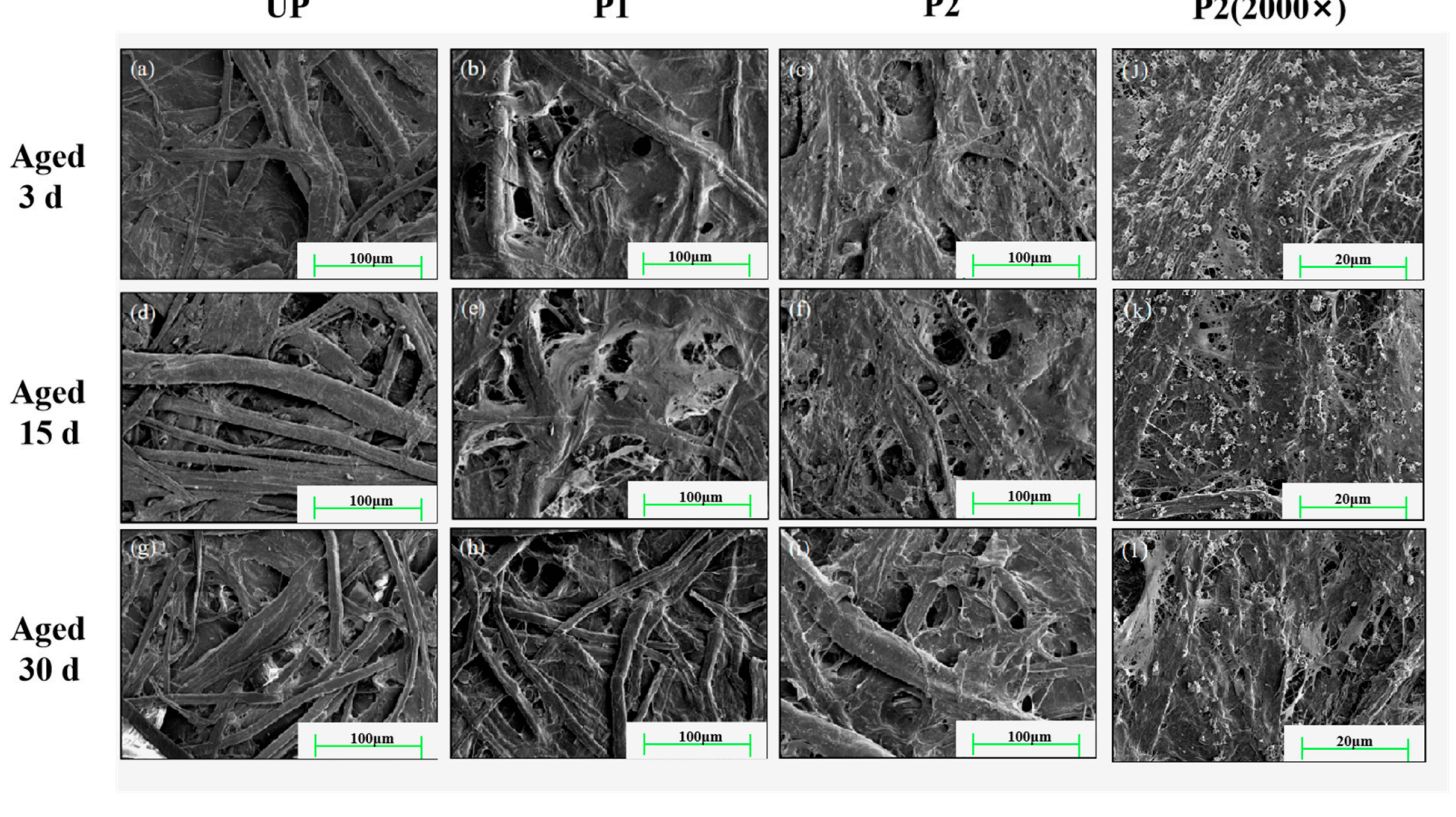
FESEM images of the paper samples during the aging process, wherein (**a**–**i**) represented 500× magnification and (**j**–**l**) represented 2000× magnification.

SEM analysis was employed to assess the distribution of the active ingredients of the repaired dispersion on paper. After aging for 3 d, the fillers on the surface of UP were observed to have diminished in quantity. However, there were no discernible alterations in the morphology of P1 and P2. The smooth KH550-BC and the rough KH550-BC/MgO were observed to lie on the surface, with the fiber structure remaining apparent (Figure 9a–c). With the extension of aging time (Figure 9d–i), the interfiber fillers of UP decreased noticeably. P1 also showed a reduction in the content of KH550-BC and an increase in the number of pores. However, the fiber morphology of the P2 had the smallest change, the aggregated structures of KH550-BC/MgO could always be recognized to be coated on the fiber surface and bridge between the fibers, and the pore changes were not obvious. It was further noted from Figure 9j,k that the MgO content gradually decreased as it played a role in acid resistance, which was in line with the weakening of its characteristic peak intensity (Figure 8c). The preceding analyses led to the reasonable conclusion that the KH550-BC/MgO is interwoven with the aging paper fiber, forming hydrogen bonds that cooperate with the anti-acidifying effect of MgO to jointly ensure the long-term stable preservation of the repaired paper.

## 3. Material and Methods

### 3.1. Material

Bacterial cellulose was supplied by Hainan Guangyu Biotechnology Co., Ltd. (Haikou, China). 3-aminopropyl triethoxysilane (KH550) and nano-MgO were both analytical grade and provided by Shanghai McLean Biochemical Technology Co., Ltd. (Shanghai, China). The paper samples were selected from an old book made of bleached hardwood pulp and published in 1972. After the natural aging process, the pH was found to be 6.45 ± 0.08, with a gram weight of 56.15 g/m^2^.

### 3.2. Preparation of KH550-BC/MgO Dispersion

In total, 1 g dry BC and 5% KH550 solution (pre-hydrolyzed in 160 mL 80% ethanol solution for 30 min) were added to a 500 mL three-mouth flask, reacted at 80 °C and 350 rpm for 4 h. Afterwards, a dispersion of KH550-BC was prepared at 40,000 rpm using a standard disperser (SKG 1246, SKG, Guangzhou, China) and then homogenized at 45 bar 5 times by a high-pressure nanohomogenizer (NanoGenizer, Genizer, Irvine, CA, USA). 

**Figure 10:**
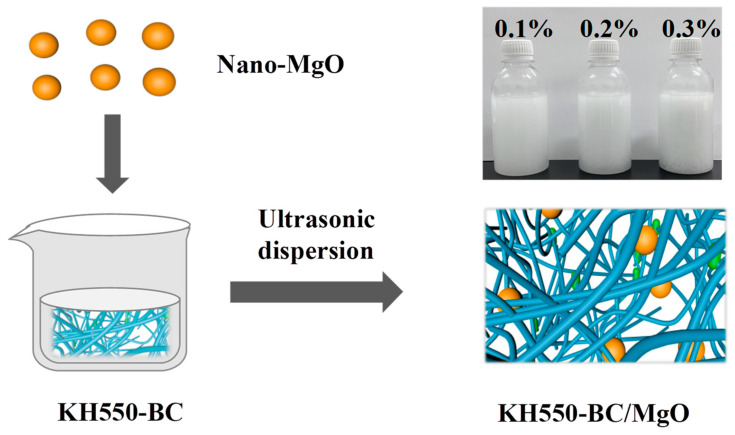
Diagram of the KH550-BC/MgO system preparation.

A specific mass of nano-MgO was added into a 0.4% concentration of KH550-BC, and the mixture was dispersed uniformly by ultrasonic dispersion (JY 99-IIDN, Scientz, Ningbo, China) for 30 min to obtain a KH550-BC/MgO dispersion. During this experiment, the mass fraction of MgO in the KH550-BC/MgO system was 0.1%, 0.2%, and 0.3%, respectively. The preparation diagram of KH550-BC/MgO is illustrated in Figure 10.

### 3.3. Artificial Accelerated Aging of Paper Samples

Aged paper that had no fractures or stains on the surface was selected for this study. Based on our previous reinforcement process [24], the paper samples were individually impregnated for 5 min in KH550-BC and KH550-BC/MgO dispersion at the same concentration (0.4%). Following impregnation, the paper samples were suspended vertically in a vacuum drying oven (BGZ-6050, Shanghai Yiheng Technology Co., Ltd., Shanghai, China) and subjected to a drying process at 50 °C for 0.5 h. They were then equilibrated with water for 24 h, according to ISO 187:1990 [52].

**Figure 11:**
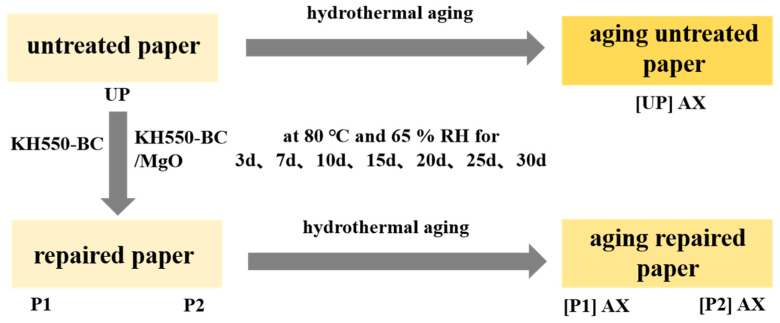
Scheme of the paper samples used and the treatments applied. X is the number of days of the hydrothermal aging test.

In accordance with ISO 5630-3:1996 [53], the untreated paper (UP), the paper treated with the KH550-BC dispersion (P1), and the paper treated with the KH550-BC/MgO dispersion (P2) were subjected to an artificial hydrothermal ageing test for specific days (3 d, 7 d, 10 d, 15 d, 20 d, 25 d, and 30 d) at 80 °C and 65% relative humidity in a constant temperature and humidity chamber (LHS-100CL, Shanghai Yiheng Technology Co., Ltd., Shanghai, China). Prior to characterization, all paper samples were suspended for 24 h at 23 °C and 50% relative humidity according to ISO 187:1990 [52]. The repair process and aging conditions of the aged paper samples are shown in Figure 11.

### 3.4. Characterization of Paper Samples

The pH value of the paper samples was determined by cold extraction according to ISO 6588-1:2021 [54]. The alkali reserve of the paper samples was determined by titration according to ISO 10716-1994 [55], and the calculation formula was as follows in Equation (1).

(1) $$X=\frac{{(V}_{2}-V_{1})c}{m}$$

where *X* is the alkali reserve of the paper samples, *V*_1_ and *V*_2_ represent the volume of NaOH consumed by the paper sample solution and blank reagent, respectively, *m* is the absolute dry mass of the paper samples, and *c* is the concentration of the NaOH standard solution.

The mechanical properties (tear index, tensile index, folding endurance, and zero-span tensile strength) testing methods had been reported in detail [17]. The tensile stress of the paper samples (4 cm × 8 mm) was determined by a material testing machine (INSTRON 3300, INSTRON, Shanghai, China) at 10 mm/min. In addition, the paper samples were placed on the Surface WCA Tester (OCA40 Micro, Dataphysics, Filderstadt, Germany) to test the surface wettability, and the drip flow of deionized water was controlled to be 8 μL.

The surface morphology of paper samples was obtained by a scanner (Epson Perfection V330, Epson, Beijing, China). In accordance with ISO 11476:2010 [56], a whiteness tester (WSB-2, Xinrui, Shanghai, China) was employed to assess the whiteness, L*, a*, and b* values of the paper samples. The chromatic aberration (ΔE*) was calculated using the following formula:

(2) $${∆E}^{*}=\sqrt{{\left({∆L}^{*}\right)}^{2}+{\left({∆a}^{*}\right)}^{2}+{\left({∆b}^{*}\right)}^{2}}$$

where ∆L* is the difference in lightness and darkness, ∆a* represents the difference in red and green, and ∆b* represents the difference in yellow and blue.

A field emission scanning electron microscope (FESEM; LEO1530VP, Zeiss, Jena, Germany) was used to assess the microscopic morphology of the paper samples. The chemical structure of the paper samples with varying degrees of aging was determined by attenuated total reflection Fourier transform infrared spectroscopy (ATR-FTIR; TENSOR27, Bruker, Ettlingen, Germany). The ATR-FTIR spectrum was captured in absorption mode with a resolution of 4 cm^−1^, a range of 4000–500 cm^−1^, and 32 scans. The crystallinity spectrum of the paper samples was obtained by a Bruker X-ray polycrystalline diffractometer (XRD, D8 ADVANCE, Bruker, Ettlingen, Germany) using a conventional BB focusing light path with a 2θ range of 5°~60°, and the crystallinity index was calculated by the Segal formula.

## 4. Conclusions

This study examined the application of BC-based composites as paper relics reinforcing agents and their potential mechanism of anti-aging. The incorporation of nano-MgO into KH550-BC, which plays an integrated role in deacidification and reinforcement, was found to be an effective method to significantly improve the durability of the paper. The experimental results demonstrate that the paper repaired by KH550-BC/MgO aged for 30 days still exhibits an adequate strength and acid-resistance ability, with approximately 30% higher mechanical properties than untreated paper and an alkali reserve of 0.25 mol/kg. ATR-FTIR and SEM analyses demonstrated that a strong interfacial binding force in coordination with sufficient alkali reserves contributed to these observed properties. These findings offer valuable insights for the long-term stable conservation of ancient books using BC, and the potential antibacterial property of repaired paper given by KH550-BC/MgO should be considered in future studies for the optimal conservation of paper relics.

## Figures and Tables

**Table 1:** The influence of MgO contents in the KH550-BC/MgO system on the pH and alkali reserve of aged paper.

Samples	Mass Fraction of MgO/%	pH	Alkali Reserve/mol kg^−1^
1	0	7.96	0.26
2	0.1	9.42	0.36
3	0.2	9.98	0.53
4	0.3	10.21	0.82

**Table 2:** Comparison of the aged paper repairing effects of two reinforcement systems.

Sample	pH	Alkali Reserve (mol/kg)	Tear Index (mN·m^2^/g)	Tensile Index (N·m/g)	Folding Endurance (Times)	Zero-Span Tensile Strength (kN/m)	Tensile Stress (MPa)
UP	6.45	0	3.48	18.70	4.50	68.30	14.27
P1	7.96	0.26	6.65	36.15	19.80	78.60	28.02
The increased ratio (%)	23.41	—	91.09	93.32	340	15.08	96.36
P2	9.42	0.36	6.60	35.44	19	77.80	27.29
The Increased ratio (%)	46.05	—	89.66	89.52	322.20	13.91	88.60

## Data Availability

Data may be shared under request.

## References

[1] Zervos S., Moropoulou A. (2006). Methodology and Criteria for the Evaluation of Paper Conservation Interventions: A Literature Review. Restaur.-Int. J. Preserv. Libr. Arch. Mater..

[2] Fan L.-t., Gharpuray M.M., Lee Y.-H., Fan L.-T., Gharpuray M.M., Lee Y.-H. (1987). Acid Hydrolysis of Cellulose. Cellulose Hydrolysis.

[3] Strlič M., Kolar J., Pihlar B., Strlič M., Kolar J. (2005). Methodology and analytical techniques in paper stability studies. Aging and Stabilisation of Paper.

[4] Strlič M., Menart E., Cigić I.K., Kolar J., de Bruin G., Cassar M. (2010). Emission of reactive oxygen species during degradation of iron gall ink. Polym. Degrad. Stab..

[5] Croitoru C., Roata I.C. (2024). Ionic Liquids as Reconditioning Agents for Paper Artifacts. Molecules.

[6] Santos A., Cerrada A., García S., San Andrés M., Abrusci C., Marquina D. (2009). Application of Molecular Techniques to the Elucidation of the Microbial Community Structure of Antique Paintings. Microb. Ecol..

[7] Magaudda G. (2004). The recovery of biodeteriorated books and archive documents through gamma radiation: Some considerations on the results achieved. J. Cult. Herit..

[8] Liang X., Zheng L., Li S., Fan X., Shen S., Hu D. (2017). Electrochemical removal of stains from paper cultural relics based on the electrode system of conductive composite hydrogel and PbO_2_. Sci. Rep..

[9] Jiang F., Yang Y., Weng J., Zhang X. (2016). Layer-by-Layer Self-Assembly for Reinforcement of Aged Papers. Ind. Eng. Chem. Res..

[10] Jia Z., Yang C., Zhao F., Chao X., Li Y., Xing H. (2020). One-Step Reinforcement and Deacidification of Paper Documents: Application of Lewis Base—Chitosan Nanoparticle Coatings and Analytical Characterization. Coatings.

[11] Li S., Tang J., Jiang L., Jiao L. (2023). Conservation of aged paper using reduced cellulose nanofibrils/aminopropyltriethoxysilane modified CaCO3 particles coating. Int. J. Biol. Macromol..

[12] Xiang Z., Liu Q., Chen Y., Lu F. (2017). Effects of physical and chemical structures of bacterial cellulose on its enhancement to paper physical properties. Cellulose.

[13] Wang Y., Luo W., Tu Y., Zhao Y. (2021). Gelatin-Based Nanocomposite Film with Bacterial Cellulose–MgO Nanoparticles and Its Application in Packaging of Preserved Eggs. Coatings.

[14] Santos S.M., Carbajo J.M., Gómez N., Quintana E., Ladero M., Sánchez A., Chinga-Carrasco G., Villar J.C. (2016). Use of bacterial cellulose in degraded paper restoration. Part I: Application on model papers. J. Mater. Sci..

[15] Gómez N., Santos S., Carbajo J., Villar J. (2017). Use of bacterial cellulose in degraded paper restoration: Effect on visual appearance of printed paper. Bioresources.

[16] Chen X., Ding L., Ma G., Yu H., Wang X., Zhang N., Zhong J. (2023). Use of bacterial cellulose in the restoration of creased Chinese Xuan paper. J. Cult. Herit..

[17] Wu X., Mou H.Y., Fan H., Yin J., Liu Y., Liu J. (2022). Improving the Flexibility and Durability of Aged Paper with Bacterial Cellulose. Mater. Today Commun..

[18] Nakayama A., Kakugo A., Gong J.P., Osada Y., Takai M., Erata T., Kawano S. (2004). High Mechanical Strength Double-Network Hydrogel with Bacterial Cellulose. Adv. Funct. Mater..

[19] He H., Teng H., An F., Wang Y.-W., Qiu R., Chen L., Song H. (2023). Nanocelluloses review: Preparation, biological properties, safety, and applications in the food field. Food Front..

[20] Zhang X., Yao J., Yan Y., Huang X., Zhang Y., Tang Y., Yang Y. (2024). Reversible Deacidification and Preventive Conservation of Paper-Based Cultural Relics by Mineralized Bacterial Cellulose. ACS Appl. Mater. Interfaces.

[21] Choo K.W., Dhital R., Mao L., Lin M., Mustapha A. (2021). Development of polyvinyl alcohol/chitosan/modified bacterial nanocellulose films incorporated with 4-hexylresorcinol for food packaging applications. Food Packag. Shelf Life.

[22] Shao W., Wu J., Liu H., Ye S., Jiang L., Liu X. (2017). Novel bioactive surface functionalization of bacterial cellulose membrane. Carbohydr. Polym..

[23] Fernandes S.C., Sadocco P., Alonso-Varona A., Palomares T., Eceiza A., Silvestre A.J., Mondragon I., Freire C.S. (2013). Bioinspired antimicrobial and biocompatible bacterial cellulose membranes obtained by surface functionalization with aminoalkyl groups. ACS Appl. Mater. Interfaces.

[24] Mou H., Wu T., Wu X., Zhang H., Ji X., Fan H., Song H. (2024). Improvement of interface bonding of bacterial cellulose reinforced aged paper by amino-silanization. Int. J. Biol. Macromol..

[25] Ahn K., Banik G., Potthast A. (2012). Sustainability of Mass-Deacidification. Part II: Evaluation of Alkaline Reserve. Restaur. Int. J. Preserv. Libr. Arch. Mater..

[26] Li Y., Wang J., Jia Z., Zhou Y., Chao X., Terigele, Li J., Li Y., Xing H. (2023). Deacidification and consolidation of brittle book paper using bacterial cellulose composite with zinc oxide nanoparticles. J. Cult. Herit..

[27] Jablonsky M., Holubkova S., Kazikova J., Botkova M., Haz A., Bajzikova M. (2013). The treatment of acid newsprint paper: Evaluation of treatment bymgo or by a mixture of MgO and methyl methoxy magnesium carbonate. Wood Res..

[28] Huang J., Liang G., Lu G., Zhang J. (2018). Conservation of acidic papers using a dispersion of oleic acid-modified MgO nanoparticles in a non-polar solvent. J. Cult. Herit..

[29] He B., Ai J., Qi S., Ren J., Zhao L., Liu C., Fan H. (2022). Highly stable nano magnesium oxide organic coatings for nondestructive protection of acidic paper documents. Prog. Org. Coat..

[30] Amornkitbamrung L., Bračič D., Bračič M., Hribernik S., Malešič J., Hirn U., Vesel A., Stana-Kleinschek K., Kargl R., Mohan T. (2020). Comparison of Trimethylsilyl Cellulose-Stabilized Carbonate and Hydroxide Nanoparticles for Deacidification and Strengthening of Cellulose-Based Cultural Heritage. ACS Omega.

[31] Wu X., Xiang Z., Song T., Qi H. (2019). Wet-strength agent improves recyclability of dip-catalyst fabricated from gold nanoparticle-embedded bacterial cellulose and plant fibers. Cellulose.

[32] Zhang X., Yan Y., Yao J., Jin S., Tang Y. (2023). Chemistry directs the conservation of paper cultural relics. Polym. Degrad. Stab..

[33] Kumar S., Chauhan V.S., Chakrabarti S.K. (2016). Separation and analysis techniques for bound and unbound alkyl ketene dimer (AKD) in paper: A review. Arab. J. Chem..

[34] Basta A., El-Saied H., Mohamed S., El-sherbiny S. (2006). The Role of Neutral Rosin-Alum Size in the Production of Permanent Paper. Restaur.-Int. J. Preserv. Libr. Arch. Mater..

[35] Wu C., Liu Y., Hu Y., Ding M., Cui X., Liu Y., Liu P., Zhang H., Yang Y., Zhang H. (2023). An Investigation into the Performance and Mechanisms of Soymilk-Sized Handmade Xuan Paper at Different Concentrations of Soymilk. Molecules.

[36] Kono H., Uno T., Tsujisaki H., Anai H., Kishimoto R., Matsushima T., Tajima K. (2020). Nanofibrillated Bacterial Cellulose Surface Modified with Methyltrimethoxysilane for Fiber-Reinforced Composites. ACS Appl. Nano Mater..

[37] Zervos S., Lejeune A., Deprez T. (2010). Natural and Accelerated Ageing of Cellulose and Paper: A Literature Review. Cellulose Structure and Properties, Derivatives and Industrial Uses.

[38] Wong K.K.Y., Richardson J.D., Mansfield S.D. (2000). Enzymatic Treatment of Mechanical Pulp Fibers for Improving Papermaking Properties. Biotechnol. Prog..

[39] Liu J., Gao S., Wang Y., Li X., Xuan C. (2013). Effect of ambient humidity on water content and mechanical properties of paper. J. Beijing Inst. Print..

[40] He B., Lin Q., Chang M., Liu C., Fan H., Ren J. (2019). A New and Highly Efficient Conservation Treatment for Deacidification and Strengthening of Aging Paper by In-situ Quaternization. Carbohydr. Polym..

[41] Zou X., Uesaka T., Gurnagul N. (1996). Prediction of paper permanence by accelerated aging I. Kinetic analysis of the aging process. Cellulose.

[42] Łojewski T., Miśkowiec P., Molenda M., Lubańska A., Łojewska J. (2010). Artificial versus natural ageing of paper. Water role in degradation mechanisms. Appl. Phys. A.

[43] Safaei M., Taran M. (2022). Preparation of Bacterial Cellulose Fungicide Nanocomposite Incorporated with MgO Nanoparticles. J. Polym. Environ..

[44] Liang G., Lu G., Zhang J., Zhen D. (2017). Study on the application of nano-magnesium oxide to the deacidification of paper cultural relics. New Chem. Mater..

[45] Hajji L., Boukir A., Assouik J., Pessanha S., Figueirinhas J.L., Carvalho M.L. (2016). Artificial aging paper to assess long-term effects of conservative treatment. Monitoring by infrared spectroscopy (ATR-FTIR), X-ray diffraction (XRD), and energy dispersive X-ray fluorescence (EDXRF). Microchem. J..

[46] Wang Y.J., Zhang X.N., Song Y., Zhao Y., Chen L., Su F., Li L., Wu Z.L., Zheng Q. (2019). Ultrastiff and Tough Supramolecular Hydrogels with a Dense and Robust Hydrogen Bond Network. Chem. Mater..

[47] Jinquan W., Yongwen M., Yan W., Chen Y. (2013). The Content of Different Hydrogen Bond Models and Crystal Structure of Eucalyptus Fibers during Beating. BioResources.

[48] Wu C., Jin C., Zhu Z., Liu P., Zhang H. (2022). Discussion on the method of detecting the crystalline structure of cellulose in paper. Fudan J. (Nat. Sci. Ed.).

[49] Ju X., Bowden M., Brown E.E., Zhang X. (2015). An improved X-ray diffraction method for cellulose crystallinity measurement. Carbohydr. Polym..

[50] Hassan R. (2016). Thermal degradation of paper: The structural changes of fibres. Egypt. J. Archaeol. Restor. Stud..

[51] Ma X., Tian S., Li X., Fan H., Fu S. (2021). Combined Polyhexamethylene Quanidine and Nanocellulose for the Conservation and Enhancement of Ancient Paper. Cellulose.

[52] (1990). Paper, Board and Pulps—Standard Atmosphere for Conditioning and Testing and Procedure for Monitoring the Atmosphere and Conditioning of Samples.

[53] (1996). Paper and Board—Accelerated Ageing Part 3: Moist Heat Treatment at 80 Degrees C and 65% Relative Humidity.

[54] (2021). Paper, Board and Pulps—Determination of pH of Aqueous Extracts—Part 1: Cold Extraction.

[55] (1994). Paper and Board—Determination of Alkali Reserve.

[56] (2010). Paper and Board—Determination of CIE Whiteness, C/2 Degrees (Indoor Illumination Conditions).

